# Dataset of the effect of sulfur dosage in solid rubber compound and vulcanizate in rubberrized asphalt properties

**DOI:** 10.1016/j.dib.2025.111461

**Published:** 2025-03-20

**Authors:** Henry Prastanto, Illah Sailah, Ono Suparno, Madi Hermadi

**Affiliations:** a PT Riset Perkebunan Nusantara, Bogor, 16151, Indonesia; b Department of Agro-industrial Technology, IPB University, Bogor, 16680, Indonesia; c Road Materials Center, Directorate of Road and Bridge Engineering, Directorate General of Highways, Ministry of Public Works, Bandung, 40294, Indonesia; d Garut Technology Institute, Garut, 44151, Indonesia

**Keywords:** Crosslink, Performance grade, Polymer modified bitumen, SIR 20, TSR 20, Vulcanization, Rubberized asphalt

## Abstract

The characteristics of natural rubber greatly influence the quality of asphalt modified with natural rubber. This research uses natural rubber of the Technical Specified Rubber (TSR) 20 type, which in Indonesia is called Standard Indonesian Rubber (SIR) 20 as the raw material. The sulfur vulcanization material is expected to increase crosslinks so that it can increase the resistance of natural rubber to hot asphalt and can improve the quality of rubberized asphalt. This research was designed by varying the sulfur dosage and vulcanization duration. The sulfur dosage used varies, namely 5; 10; 15; 20 parts per hundreed rubber (phr). The duration of vulcanization time at 140 °C for each dose of sulfur is 0;30;60;90;120 min. The compound or vulcanisate is then mixed into the asphalt at a temperature of 160–170 °C with a dose of 2 % rubber and 98 % asphalt. Based on the research results, it is known that increasing the sulfur dosage and vulcanization time duration produces rubber that is more resistant to oil through testing for solubility and swelling in toluene. The unvulcanized compound dissolves in toluene, while the vulcanizate swelling ratio decreases with increasing sulfur dosage (790 to 482 %). Increasing the sulfur dosage and the duration of vulcanization time produces harder rubber so that the duration of mixing into asphalt increases from 1.5 h to 5.5 h. The addition of sulfur dosage and vulcanization time duration can increase the original Performance Grade (PG) and PG after Rolling Thin Film Oven Test (RTFOT) when compared to original asphalt. Original PG of rubberized asphalt obtained from this research is 69.8 to 90.4 °C compared to original asphalt PG which is only 66.7 °C. PG after RTFOT was obtained between 60.3 to 88.4 °C compared to original asphalt PG which was only 64 °C. This research data can be useful in the design of rubber compounds and vulcanizates which can be selected by considering process engineering, processing machines and economic aspects in the production of rubberized asphalt.

Specifications Table

**Table:** 

Subject	Chemical Engineering.
Specific subject area	Agro Industrial Technology, Polymer Technology, Rubber Technology, Asphalt Technology.
Type of data	Table and Graph. Raw, Analyzed.
Data collection	Swelling and solubility data were obtained by dissolving or immersing the rubber in toluene for 72 h at room temperature. The swelling ratio was calculated from the additional weight of the vulcanisate. Mixing time data was obtained by recording the time the rubber dissolved into the asphalt until it was homogeneous using a high shear mixer. Original PG and RTFOT PG data were obtained by asphalt testing using a Dynamic Shear Rheometer (DSR) machine and rotary oven. Softening point data was obtained by testing rubberized asphalt with a ring and ball test equipment. Rubberized asphalt penetration data was obtained by testing rubberized asphalt using a laboratory penetration test equipment. The viscosity of rubberized asphalt was obtained by testing the viscosity of rubberized asphalt with a Brookfield viscosity tester.
Data source location	Laboratory of PT Riset Perkebunan Nusantara, Bogor, Indonesia, and Laboratory of PT Bintang Djaja, Cilacap, Indonesia. Latitude and longitude for collected samples/data: 6°35′16.6"S 106°48′17.7"E and 7°42′21.7"S 108°59′51.7"E.
Data accessibility	Repository name: Mendeley Data Data identification number: 10.17632/wnwykdkwx3.2. Direct URL to data: https://data.mendeley.com/datasets/wnwykdkwx3/2
Related research article	- .

## 1 Value of the Data

• The dataset provides information about the influence of sulfur dosage in rubber and the duration of vulcanization time on rubber swelling in organic solvents and the properties of rubberized asphalt produced, where currently this information is still very limited
• The dataset is useful for improving the quality of rubberized asphalt and reducing production costs which is currently being developed by increasing the resistance of rubber in hot asphalt and reducing the dosage of rubber in asphalt
• The dataset provides information for further research and development regarding rubber formulation technology, rubberized asphalt production process engineering and the design of tools or machines used.

## 2 Background

Polymer modified asphalt technology has been widely used to improve the quality of asphalt roads where Styrene Butadiene Styrene (SBS), rubber powder from used tires [1] is generally used. The use of natural rubber asphalt in Indonesia has begun to be used at a dose of 5–7 % because it has a high softening point, is elastic and is stickier [[2], [3]]. The first limitation of using natural rubber as an asphalt additive is that the price of asphalt modified with natural rubber is more expensive than the synthetic polymer SBS because it uses a relatively higher dose of natural rubber. The second is that the quality of asphalt modified with natural rubber is still lower than asphalt modified with the synthetic polymer SBS. And third, if the dose is higher, the mixing time will be longer, resulting in natural rubber being easily damaged because it is degraded by heat. The durability of rubber in asphalt is greatly influenced by the degree of cross-linking in the rubber due to the vulcanization reaction [4]. Vulcanization reactions are generally carried out with sulfur reagents at high temperatures [5]. Natural rubber with low cross-linking is easy to mix in asphalt but is easily degraded in hot asphalt [6]. Very high cross-linking in rubber causes the rubber to lose elasticity and become a hard [7]. Hard natural rubber will be difficult to dissolve in asphalt so it will be dispersed and then swell in the asphalt [[8], [9]]. The elastic properties of rubber are still needed to increase the elasticity of rubberized asphalt, so a sulfur dosage and vulcanization time duration must be sought that provide resistance to hot asphalt but remain elastic.

## 3 Data Description

The SIR 20 used in this research was produced by PTPN I regional 7. The results of the SIR 20 quality testing are presented in Table 1. The SIR 20 testing was carried out in accordance with the Indonesian National Standard (SNI 1903:2017). The SIR 20 used based on the research results meets the requirements in accordance with the SIR 20 specifications. The main parameters used in testing are Po and PRI which indicate that the natural rubber used has a standard molecular chain length and has good heat resistance. f P0 is low it will result in low PG, low softening point and low viscosity. If the PRI is low, the rubber will easily be degraded by heat, as a result the rubber will be easily damaged when mixed with hot asphalt.

**Table 1:** Data on test results of the rubber (SIR 20).

Properties of Natural Rubber	Method	Standard	Result
Unaged Plasticity (Po), Min	SNI ISO 2930:2013	30	40
Aged Plasticity (Pa), Min	SNI ISO 2930:2013	–	28,5
Plasticity Retention Index (PRI) %, Min	SNI ISO 2930:2013	50	71,3
Dirt content, %, Max	SNI ISO 249:2015	0,18	0,09
Volatile Matter content, %, Max	SNI 8356:2017	0,08	0,23
Nitrogen content, %, Max	SNI ISO 1565:2016	0,60	0,42
Ash content, %, Max	SNI ISO 247:2014	1,0	0,31

The asphalt used in this research is asphalt penetration grade 60/70 produced by PT Pertamina. The asphalt properties test results are presented in Table 2. Asphalt penetration still meets the asphalt penenetration grade 60/70 requirements. The original PG and residual RTFOT PG were 66.7 °C and 64 °C respectively and this data was used as a based line to see the increase in PG with the addition of rubber.

**Table 2:** Data on test results of the original asphalt.

Properties of Asphalt	Method	Result
Viscositas @135, cps	SNI 06-6441:2000	430
Penetrasi, dmm	SNI 2432:2011	65
Softening point, °C	SNI 2434:2011	52
Performance Grade, Original, °C	SNI 03-6442:2000	66,7
Performance Grade, RTFOT, °C	SNI 03-6442:2000	64,0

This research was carried out by mixing sulfur into SIR 20 with varying sulfur doses, namely 5;10;15;20 phr. This sulfur dose is an intermediate dose between soft or elastic rubber (below 5 phr) and hard or inelastic rubber (above 20 phr). The difference of 5 phr was chosen so that the difference in dose was easier to observe and not too many samples were taken. Each sample uses the consecutive codes A;B;C;D. Each sample was vulcanized at a temperature of 140 °C in an oven for different times duration, namely 0;30;60;90;120 min to determine the effect of vulcanization time duration on the quality of rubber and rubberized asphalt. Time 0 min or not vulcanized was used as a control. The vulcanization time duration was chosen because soft rubber usually has a vulcanization time of under 30 min while hard rubber takes up to 2 h. The time difference interval is taken to be 30 min so that the time difference has begun to be observed and the amount of data taken is sufficient (Table 3).

**Table 3:** Treatment for sulfur variations and duration of vulcanization.

Duration of Rubber Vulcanization at 140 °C, minutes	Dosage of Sulfur, phr			
	A	B	C	D
0	5	10	15	20
30	5	10	15	20
60	5	10	15	20
90	5	10	15	20
120	5	10	15	20

Rubber that has been mixed with sulfur and vulcanized, then made into powder is analyzed for its chemical composition as presented in Table 4. Adding a dose of sulfur will increase the percentage of material extracted in acetone. Increasing the vulcanization time will reduce the material extracted in acetone. Other compositions such as polymer content, carbon content and ash content relatively do not differ much due to increasing sulfur dosage or increasing vulcanization time

**Table 4:** Chemical composition of rubber compound and rubber vulcanizate.

Sample Code	Duration of Rubber Vulcanization	Acetone Extract Content	Polymer Type	Polymer Content	Carbon Content	Ash Content
	minutes	%		%	%	%
	0	8,46	IR	86,34	0,41	4,79
	30	5,74	IR	87,80	1,25	5,74
A	60	4,84	IR	89,43	0,94	4,84
	90	3,69	IR	90,62	0,82	3,69
	120	3,57	IR	89,20	2,39	3,57
	0	7,57	IR	86,93	0,43	4,97
	30	6,32	IR	87,07	1,90	5,03
B	60	6,20	IR	88,31	0,73	4,77
	90	4,30	IR	89,66	1,11	4,94
	120	4,33	IR	89,24	1,82	4,62
	0	9,31	IR	85,94	0,21	4,54
	30	7,30	IR	87,56	0,52	4,63
C	60	6,14	IR	88,51	0,83	4,53
	90	5,29	IR	88,68	1,41	4,63
	120	3,18	IR	91,33	1,00	4,50
	0	9,86	IR	83,78	0,30	4,06
	30	7,30	IR	87,63	0,52	4,55
D	60	7,77	IR	85,94	1,95	4,35
	90	7,95	IR	84,67	2,87	4,52
	120	8,19	IR	83,78	3,60	4,44

IR : Isoprene Rubber

**Fig. 1:**
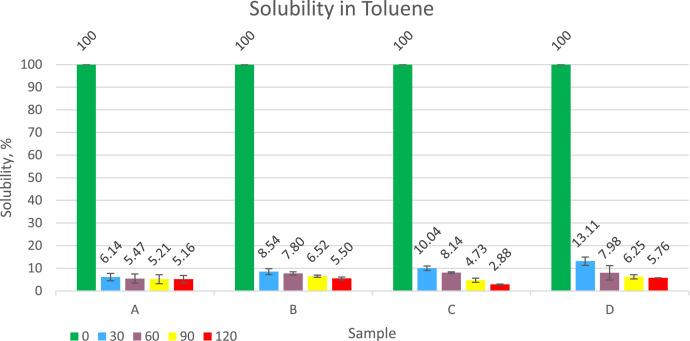
Graph of the solubility of rubber into toluene.

**Fig. 2:**
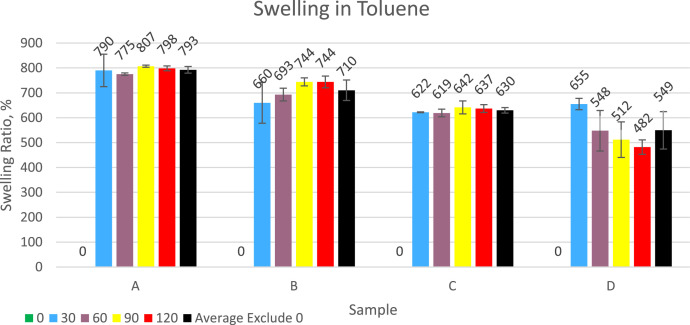
Graph of swelling ratio of rubber in toluene.

Compounds and vulcanized powder before being mixed into asphalt are tested for solubility and swelling in toluene. The more the rubber swells, the more it will absorb the light fraction oil in the asphalt, causing the asphalt to stiffen and crack more easily. The test results are presented in Fig. 1, Fig. 2. The solubility of the compound that has not been vulcanized is 100 % and can be said to dissolve completely. For all samples that had been vulcanized from 30 min to 120 min, the solubility varied from 2.88 % to 13.11 %. The addition of the sulfur dose according to research data will be followed by an increase in the solubility of the material in toluene. The increase in vulcanization time duration is followed by a decrease in the percentage of material dissolved in toluene. Rubber swelling occurs due to the entry of toluene into the rubber matrix where the rubber powder sample is quite porous. Swollen rubber will be softer and more easily dispersed into asphalt. Based on research data, the addition of sulfur causes rubber vulcanization to become harder and less easy to swell in toluene. Increasing the duration of vulcanization time does not consistently affect the swelling because samples A and C tend not to differ much, sample B tends to increase the swelling while for sample D the swelling tends to decrease. The swelling ratio values ​​of the samples varied from 482 % to 807 %.

Vulcanized powder with a maximum size of 12 mesh is mixed into the asphalt. Compounds that have not been vulcanized tend to be sticky so they cannot be made into powder but can be cut larger, with a sample cut size of length or width or a maximum diameter of 5 mm. The dosage of compound or vulcanisate in the asphalt is 2 %. The compound mixing time is longer than for some vulcanized powders because the size of the vulcanized powder is smaller when mixed. The addition of sulfur and vulcanization time to rubber powder causes an increase in the duration of mixing time. The sample that took the longest to mix (5.5 h) was the hardest sample, namely D with a vulcanization time of 120 min. The longer the mixing time, the higher the costs, the lower the productivity and the more rubber damage due to heat. The duration of mixing time is presented in Table 5.

**Table 5:** Time duration for mixing rubber and asphalt.

Duration of Rubber Vulcanization at 140 °C, minutes	Duration of Mixing, hours			
	A	B	C	D
0	2,0	2,0	2,0	2,0
30	1,5	1,5	1,5	2,5
60	1,5	1,5	1,5	3,5
90	2,0	2,0	2,5	4,0
120	3,0	3,0	4,0	5,5

**Fig. 3:**
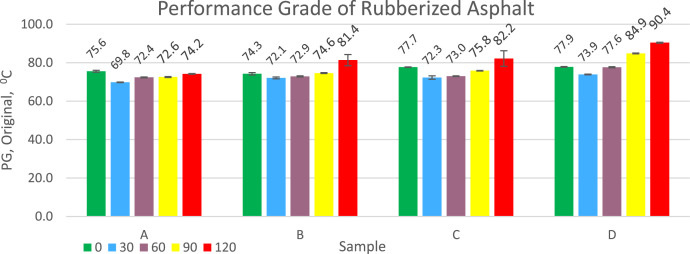
Graph of Performance Grade (PG) Original test results of rubberized asphalt ageing.

Performance grade indicates the asphalt's resistance to loads at a certain temperature. The higher the PG, the more resistant it is to receiving loads at high temperatures. The effect of asphalt PG on the properties of asphalt on roads is: fist, in terms of resistance to extreme weather, asphalt with a higher PG has a higher softening point so it is stronger in facing extreme weather. The second is in terms of suitability for heavy traffic where asphalt with a higher PG is more suitable for heavy traffic. Rubberized asphalt PG data for each treatment is presented in Fig. 3. Adding a dose of sulfur to the compound that has not been vulcanized or vulcanized powder can increase the PG of rubberized asphalt. Increasing the duration of vulcanization time can also increase the PG of rubberized asphalt. A different thing happened when adding rubber in compound form to asphalt, some samples actually had a higher PG value for rubberized asphalt when compared to vulcanized powder. The PG of original rubberized asphalt ranges from 69.8 °C to 90.4 °C and this is higher than the PG of pen 60/70 asphalt which has not had rubber added which is 66.7 °C.

**Fig. 4:**
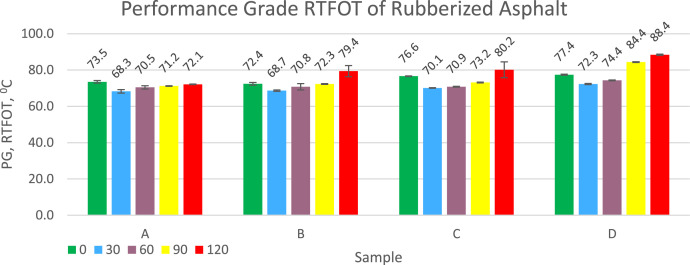
Graph of Performance Grade (PG) RTFOT test results of rubberized asphalt.

PG RTFOT shows the strength of asphalt to withstand loads after undergoing aging treatment in a rotary oven. This conditioning is to describe the durability of asphalt during storage, mixing with aggregate until installation during road construction work. The low reduction from PG original to PG after RTFOT indicates that the rubberized asphalt is not easily degraded by heat during the process. The PG RTFOT value of rubberized asphalt for each treatment is presented in Fig. 4. Adding a dose of sulfur to the compound that has not been vulcanized or vulcanized powder can increase the PG RTFOT of rubberized asphalt. Increasing the duration of vulcanization time can also increase the PG RTFOT of rubberized asphalt. A different thing happened when adding rubber in compound form to asphalt, some samples actually had a higher PG value for RTFOT rubberized asphalt when compared to vulcanized powder. The PG of RFTOT rubberized asphalt ranges from 68.3 °C to 88.4 °C and this is higher than the PG of pen 60/70 asphalt without rubber added which is 64 °C.

**Fig. 5:**
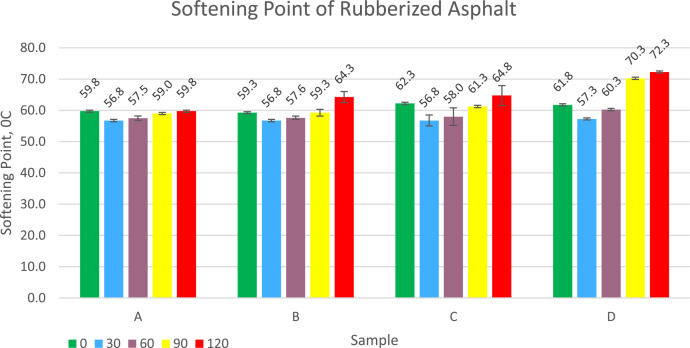
Graph of Softening Point of Rubberized Asphalt.

Asphalt softening point data for each treatment varies from 56.8 °C to 72.3 °C. Complete data is presented in the graph in Fig. 5. This value is higher than original asphalt which is only 52 °C, so this rubberized asphalt is more resistant to high temperatures. Asphalt that is too soft can easily cause deformation and grooves. Asphalt with a high softening point can produce mixtures that have small permanent deformation or high Marshall Stability. Increasing the dose of sulfur increases the softening point and increasing the duration of vulcanization time also increases the softening point for each dose of sulfur. Rubberized asphalt from compound has a higher softening point than rubberized asphalt from compound

**Fig. 6:**
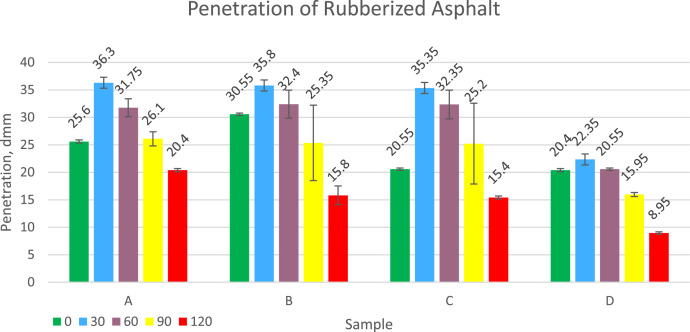
Graph of Penetration of Rubberized Asphalt.

Rubberized asphalt penetration indicates the hardness of the asphalt. The smaller the penetration, the harder the rubber asphalt is. Asphalt with low penetration is more stable because it is stiff and can withstand heavy traffic loads without experiencing permanent deformation. In addition, asphalt with low penetration has a high surface roughness, is rougher, thereby increasing vehicle safety. Penetration data varies between 8.95 to 36.3 dmm, lower than original asphalt which has a value of 65 dmm. The test result data is presented in Fig. 6. The test result data shows that the higher the sulfur dosage temperature, the harder the rubberized asphalt will be. Rubber asphalt from rubber compounds is shown to be harder than some rubberized asphalt from vulcanization.

The viscosity of rubberized asphalt varies between 523 cp to >2000 cp (Table 6), this data is higher than original asphalt which is 430 cp. Asphalt with a low viscosity tends to flow like a lubricant, so it cannot adhere well to the aggregate. Asphalt with high viscosity is difficult to compact with aggregate, so the asphalt will not be uniform. The addition of sulfur dosage increases the viscosity. Increasing the duration of vulcanization time also increases viscosity. The viscosity of rubberized asphalt from rubber compounds is higher than that of rubberized asphalt from vulcanization.

**Table 6:** Viscosity of rubberized asphalt.

Duration of Rubber Vulcanization at 140 °C, minute	Viscosity at 135 °C, cps			
	A	B	C	D
0	750	734	860	864
30	523	559	590	592
60	601	615	674	844
90	652	658	814	1562
120	740	835	909	>2000

The addition of sulfur in rubber compounds or vulcanizates as an asphalt additive has been proven to improve the quality of rubberized asphalt. Using only 2 % rubber, several samples have been obtained that meet the criteria for asphalt with a minimum PG value of 70. With this discovery, the production costs of rubber asphalt can be reduced, thereby providing economic benefits. The use of natural rubber as a renewable natural material also provides added value for the environmental aspect.

The selection of the dose and vulcanization time to be used in the next stage or implementation is of course accompanied by criteria according to technical aspects (availability of production process machines, process costs (vulcanization time and mixing time), and desired rubberized. asphalt properties.

## 4 Experimental Design, Materials and Methods

Research on the preparation and testing of rubber was carried out at the experimental factory and rubber testing laboratory of PT Riset Perkebunan Nusantara, Bogor. Mixing and testing of rubberized asphalt was carried out at laboratory of PT Bintang Djaja, Cilacap.

### 4.1 Rubber preparation

The 600 gr of SIR 20 is mixed with sulfur and rubber chemicals such as accelerators, activators and anti-oxidants using an open mill (two roll mill rubber mixing machine) with a capacity of 1 kg. The sulfur doses used were varied with doses of 5; 10; 15; 20 phr (30;60;90;120 gr) and were given sample codes A; B; C; D. Mixing was carried out for 12 min at 60 °C.

4 types of rubber compound with a thickness of 6 mm are then vulcanized in an automatic oven equipped with a blower (so that the air temperature is homogeneous) at a temperature of 140 °C with a vulcanization time duration of 0; 30; 60; 90; 120 min. Samples with 0 min vulcanization mean that they were not vulcanized in an oven. The vulcanized rubber is then reduced in size to a maximum size of 12 mesh. This sample preparation was carried out in two repetitions.

### 4.2 Rubber properties testing

The composition of the rubber compound and vulcanisate was then measured in stages starting with extraction in acetone at 70 °C for 16 h using 1 set of Soxhlet extraction equipment. The material that is not extracted is then continued with thermogravimetric testing at 450 °C to determine the levels of polymer and carbon (ASTM D297-15). Determination of ash content is based on ashing in a kiln at a temperature of 550 °C. The polymer type testing method is based on analysis using an infrared spectrophotometer (ASTM D297-15)

Apart from testing the chemical composition, the rubber compound and vulcanizate were also tested for solubility and swelling in toluene. 1 gram of rubber powder that has been wrapped in filter paper is soaked in toluene for 72 h. Rubber swelling was calculated based on the initial sample weight divided by the wet sample weight immediately after immersion. The calculation formula is as follows

%Swelling=((wetweight−initialweight)/(initialweight))×100%

### 4.3 Rubberized asphalt properties testing

Rubber vulcanisate is mixed into 250 grams of asphalt with a dosage of 2 % rubber vulcanisate at a temperature of 160–170 °C using a high shear mixer with a mixer rotation speed of 2500–3000 rpm followed by homogenization at a mixer speed of 7500 rpm for 30 min. The time required for mixing until homogeneous is recorded as the mixing time of asphalt and rubber.

The rubberized asphalt obtained was then tested for its rubberized asphalt characteristics based on the Indonesian National Standard (SNI) which refers to ASTM and AASHTO, namely PG, PG RTFOT with Dynamic Shear Rheometer (DSR) according to SNI 03-6442-2000 After conditioning, the sample is then placed in the DSR and testing begins where the testing temperature starts at 45 °C. The sample was then loaded by stretching it with an oscillation speed of 10 rad/sec. The temperature when the strain value is 1 KPa is the PG (original) value of the sample. The PG (RTFOT) sample was tested after accelerated aging of a thin layer of asphalt in a rotary oven at a temperature of 1630C for 75 min. The temperature when the strain value is 2 KPa is the PG RTFOT value of the sample.

Softening point testing was obtained by testing rubber asphalt with a ring and ball test equipment(SNI 2434-2011).Penetration data was obtained by testing rubberized asphalt using a laboratory penetration test equipment (SNI 2456:2011), and viscosity was carried out using a Brookfield viscometer at a temperature of 135 °C (SNI 06-6441-2000).

## Limitations

Not applicable.

## Ethics Statement

The dataset described in this article does not involve any human subjects, animal experi- ments, or data collected from social media platforms.

## CRediT Author Statement

**Henry Prastanto:** Conceptualization, Methodology, Validation, Resources, Writing – review & editing; **Illah Sailah:** Conceptualization, Supervision, Validation, review & editing; **Ono Suparno:** Validation, review & editing; **Madi Hermadi:** Conceptualiization, Methodology, Validation, review & editing.

## Data Availability

Mendeley DataDataset of Properties of Asphalt Mixed with Vulcanized Natural Rubber (Original data).
